# P2Y2R Signaling Is Involved in the Onset of Glomerulonephritis

**DOI:** 10.3389/fimmu.2018.01589

**Published:** 2018-07-16

**Authors:** Laura Rennert, Stefan Zschiedrich, Lukas Sandner, Björn Hartleben, Sanja Cicko, Cemil Korcan Ayata, Charlotte Meyer, Andreas Zech, Robert Zeiser, Tobias B. Huber, Marco Idzko, Florian Grahammer

**Affiliations:** ^1^Department of Medicine IV, Medical Center – University of Freiburg, Faculty of Medicine, University of Freiburg, Freiburg, Germany; ^2^Department of Pneumology, University Medical Center Freiburg, Freiburg, Germany; ^3^Department of Hematology, Oncology and Stem Cell Transplantation, University Medical Center Freiburg, Freiburg, Germany; ^4^III. Department of Medicine, University Medical Center Hamburg-Eppendorf, Hamburg, Germany; ^5^BIOSS Center for Biological Signalling Studies, Albert-Ludwigs-University, Freiburg, Germany; ^6^Division of Pulmonology, Department of Medicine II, Medical University Vienna, Vienna, Austria

**Keywords:** glomerulonephritis, leukocytes, purinergic receptors, P2Y2, adenosine-5’-triphosphate, animal model

## Abstract

Endogenously released adenosine-5’-triphosphate (ATP) is a key regulator of physiological function and inflammatory responses in the kidney. Genetic or pharmacological inhibition of purinergic receptors has been linked to attenuation of inflammatory disorders and hence constitutes promising new avenues for halting and reverting inflammatory renal diseases. However, the involvement of purinergic receptors in glomerulonephritis (GN) has only been incompletely mapped. Here, we demonstrate that induction of GN in an experimental antibody-mediated GN model results in a significant increase of urinary ATP-levels and an upregulation of P2Y2R expression in resident kidney cells as well as infiltrating leukocytes pointing toward a possible role of the ATP/P2Y2R-axis in glomerular disease initiation. In agreement, decreasing extracellular ATP-levels or inhibition of P2R during induction of antibody-mediated GN leads to a reduction in all cardinal features of GN such as proteinuria, glomerulosclerosis, and renal failure. The specific involvement of P2Y2R could be further substantiated by demonstrating the protective effect of the lack of P2Y2R in antibody-mediated GN. To systematically differentiate between the function of P2Y2R on resident renal cells versus infiltrating leukocytes, we performed bone marrow-chimera experiments revealing that P2Y2R on hematopoietic cells is the main driver of the ATP/P2Y2R-mediated disease progression in antibody-mediated GN. Thus, these data unravel an important pro-inflammatory role for P2Y2R in the pathogenesis of GN.

## Introduction

Glomerulonephritis (GN) is an immunological disease and a major cause of dialysis-dependent end stage kidney disease. Rapidly progressive glomerulonephritis (RPGN) is a subgroup of this entity, which is characterized by a rapid loss of renal function within days as well as proteinuria and glomerular hematuria. RPGN is associated with anti-glomerular basement membrane (GBM) antibodies or caused by other diseases such as ANCA associated vasculitis or IgA nephropathy. It represents one of the diagnostic and therapeutic emergencies in nephrology (1).

Pathologically, it is defined by crescents in at least 50% of glomeruli. Current therapy is composed of intense immunosuppression using plasmapheresis, high-dose steroids, and cyclophosphamide, with frequent serious adverse effects and only limited renal survival (1).

Endogenous nucleotides such as adenosine-5′-triphosphate (ATP), uridine-5′-triphosphate (UTP), or uridine-5′-diphosphate are released from intracellular storage pools into the extracellular compartment by multiple celltypes under inflammatory or hypoxic conditions (2–4). Once in the intracellular space, extracellular nucleotides function as damage-associated molecular patterns (DAMPs) *via* binding to P2 purinergic receptors (P2Rs), which can be subdivided into metabotropic P2Y (P2Y1, P2Y2, P2Y4, P2Y6, P2Y11–P2Y14) and ionotropic P2X receptors (P2X1–P2X7) (3, 4). The expression of functional P2R has been shown for both inflammatory and structural cells of the kidney (such as mesangial cells, podocytes) (5, 6). Activation of P2Rs on these cells is associated with a broad range of cellular responses including migration, release of chemokines or cytokines, production of reactive oxygen species, induction of apoptosis, or chlorid secretion (4, 5, 7–9). In addition, compelling evidences point to a role of purinergic receptor signaling in the pathogenesis of acute and chronic inflammatory renal diseases (5, 6, 10, 11).

Previously, it has been demonstrated that the expression of P2X7R is upregulated during GN (12) and that P2X7R-deficiency is associated with attenuated renal injury in experimental GN (13). Furthermore, animals lacking the P2Y1R were partially protected against renal capillary loss, glomerulosclerosis, and fibrosis (14). In a mouse model of mesangial proliferative glomerolonephritis, unselective blocking of P2R is linked to attenuation of renal injury/damage (15).

The functional expression of P2Y2 receptors, which are preferably activated by ATP or UTP, has been confirmed on different inflammatory cells including neutrophils, eosinophils, dendritic cells, as well as structural kidney cells such as podocytes and mesangial cells (3, 4, 15, 16). Recently, an involvement of the P2Y2R in chronic kidney disease progression as well as in Lithium (Li)-induced polyuria, Na+ excretion, and cyst growth has been demonstrated (17–20).

However, the precise role of P2Y2 receptors in the pathogenesis of GN is still unknown. In this study, we investigated the role of the ATP/P2Y2R-axis in an experimental model of nephrotoxic serum (NTS)-induced RPGN (21). We show that GN is associated with increased ATP-urine levels and functional upregulation of the P2Y2R. Decreasing exogenous ATP-levels by either treating wild-type (WT) animals with Apyrase and a broad P2R-antagonist or genetic depletion of P2Y2R are associated with better renal function, reduced proteinuria, and decreased histologic glomerular injury scores compared to placebo-treated mice.

## Materials and Methods

### Mice

P2Y2R-deficient and wild-type animals (both on a C57BL/J6 background) were bred under specific pathogen-free conditions, a 12 h day/night cycle and free access to rodent chow and water. All experiments were approved by the local animal ethics committee (Regierungspraesidium Freiburg).

### Animal Model of NTS-Driven GN and Treatment

Mice were given a retroorbital i.v. injection of either NTS or vehicle under ligth isoflurane anesthesia on three consecutive days with a total volume of 50 µl day 1 and 15–18 µl/g bodyweight on days 2 and 3 as previously described (22).

In the same set of experiments, mice received an i.p. injection of either suramin, apyrase, or vehicle in a total volume of 200 µl at day 1 and 100 µl at days 3, 5, 7, and 9.

Urine samples were collected at days 0, 4, 5, 6, 7, 8, and 9 to measure urinary ATP-, albumin, and creatinine levels. Blood was drawn at days 4, 6, and 10 to detect serum creatinine and albumin levels and to isolate blood leukocytes, respectively. For the analysis of P2R expression on whole kidneys, glomeruli and sorted podocytes/non-podocytes as well as for histological scoring part of the animals were killed at days 4, 6, or 10.

### Generation of Bone Marrow Chimera

Wild-type or P2Y2R^−/−^ recipients (both C57Bl/6) were given 5 × 10^6^ wt or P2Y2R^−/−^ BM cells (C57Bl/6) intravenously after bone-marrow lethal irradiation with 900 cGy (2 × 450 cGy) as previously described (23). The following donor/recipient pairs were combined: wt = >wt, P2Y2R^−/−^ = >wt (hematopoietic system: P2Y2R^−/−^), wt = > P2Y2R^−/−^ (nonhematopoietic system: P2Y2R^−/−^), P2Y2R^−/−^ = > P2Y2R^−/−^.

### Albumin, Creatinine, and ATP-Measurement in Urine and Serum

Urinary albumin and creatinine concentration were determined using the Microfluoral (Progen Biotechnik GmbH, Heidelberg, Germany) and an enzymatic Creatinine-Kit (Laboratory and Technology, Berlin, Germany), respectively (24). ATP levels in urine were measured using ATPLite kit (Perkin Elmer, Waltham, MA, USA), as previously described (25, 26).

Serum creatinine was also measured with the Creatinin-Kit (Labor und Technik, Berlin, Deutschland) and serum urea with the Urea-Kit (Labor und Technik, Berlin, Deutschland).

### Preparation of Kidneys

The kidneys were perfused with 50 µl Dynabeads in 10 ml HBSS, diameter 4.5 µm, at 37°C through the renal arteries. After homogenization with a scalpel blade, 2 ml digest solution [60 mg Collagenase 1A C5894 (Sigma) 1 mg/ml, 2 mg DNAse (Applichem) 100 U/ml, 60 ml HBSS] were added for both kidneys and the homogenate was put on a shaker at 37°C for 15 min. A filter with a pore size of 100 µm and 1× HBSS was used. The filtered solution was centrifuged with 2,000 rounds per minute at 4°C. The pellet was resuspended in HBSS and with a magnet at the tube wall washed until there were no residues of tubules under the microscope.

### Podocyte Preparation

The podocyte preparation was performed as previously described (27). Briefly, 4 ml Dynabead solution was used per kidney. This was followed by an additional perfusion with collagenase solution [1 mg/ml Worthington Collagenase Type II (Stock: 320 U/mg), 1 mg/ml Pronase E (Sigma P6911), 50 U/ml DNAse (Applichem A3778)]. The kidneys were digested and incubated with 2 ml digestion enzyme mix on the thermomixer at 37°C and 1,400 rounds per minute for 40 min. Re-suspending with a 27 G cannula and vortexing every 10 min were applied. The probe was checked with a fluorescence microscope.

The Dynabeads were then removed with a magnet. A filter (pore size 40 µm) and further washing with 10 ml HBSS were performed to generate single cells. The resultant wash through was centrifuged at 1,500 *g* at 4°C and resolved in 0.5 ml of HBSS with 0.1% BSA and 1 µg/ml 4′6-diamidino-2-phenylindole (DAPI) for subsequent viability testing. 5 µl EDTA per 1 ml suspension were added to prevent cell clogging. A MoFlo Cell Sorter (Beckman Coulter, Krefeld, Deutschland) was used with a wavelength of 380 nm (80 mW), 80 PSI for DAPI-negative and 488 nm (200 mW), 60 PSI for GFP-negative cells at 20°C.

### Isolation of Blood Leukocytes

Blood leukocytes from NTS or vehicle exposed animals were isolated as previously reported (25). Red blood cells were hemolysed by using Ery-Lysis-Buffer [8.25 g NH4Cl (155 mM), 1.0 g KHCO3 (100 mM), 0.0372 g EDTA (0,1 mM), Aqua dest. ad 1 l, pH 7.3] in 10 ml at 4°C for 20 min and centrifuged again at 1,500 *g* at 4°C for 3 min, followed by washing steps with PBS. The remaining cell pellet was used for qTPCR as described below.

### Real-Time RT-PCR

Total RNA was isolated from cell pellets or homogenized kidney tissue, isolated glomeruli, podocoytes, non-podocytes, or isolated blood leukocytes using RNeasy mini-kits (Qiagen, Hilden, Germany) as previously described (25, 28). Reverse transcription was performed using Stratascript reverse transcriptase (Stratagene, La Jolla, CA, USA) and random primers (Invitrogen, Karlsruhe, Germany). Quantitative PCR was performed with Taqman Universal PCR Mastermix (Applied Biosystems, Foster City, CA, USA) and pre-formulated primers and probe mixes (Assay on Demand, Applied Biosystems), as previously described (23, 25). PCR conditions were 2 min at 50°C, 10 min at 95°C, followed by 45 cycles of 15 s at 95°C and 60°C for 1 min using a thermal cycler (iCycler, Biorad, Hercules, CA, USA). PCR amplification of the housekeeping gene encoding β2-microglobulin (β2M) was performed during each run for each sample to allow normalization between samples.

### Histology

Removal of the kidney was performed without prior flushing with PBS to allow assessment of capillary thrombi. The samples were fixed in 4% paraformaldehyde in PBS on a shaker at 4°C for 48 h. Processing of samples overnight in paraffin was carried out automatically with a Histokinette (Leica, Wetzlar, Germany). 3 µm thin sections were made using a microtome and sections were subsequently stained at the Institute of Pathology using a staining robot.

### Statistical Analysis

If not stated otherwise, the statistical significance of differences between samples was calculated using ANOVA, followed by Bonferroni comparison test. Differences were considered significant if *P* < 0.05.

## Results

### GN Induces an Increase in ATP-Levels in the Urine

**Figure 1:**
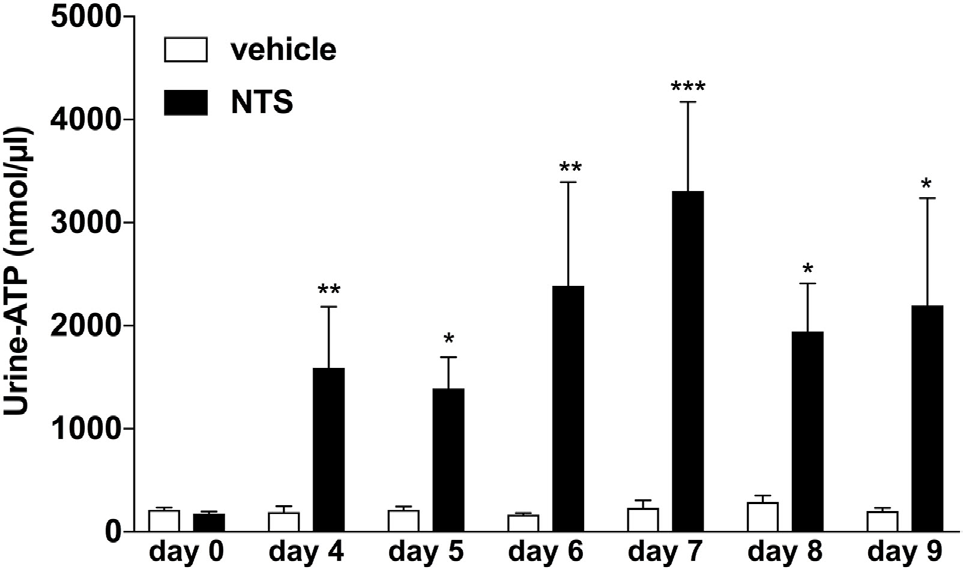
Functional analysis of urine-adenosine-5’-triphosphate (ATP) concentrations following nephrotoxic serum (NTS)-driven glomerulonephritis (GN). C57BL/6 mice received either vehicle (PBS) or (NTS solved in PBS) intravenously on days 1, 2, and 3 to induce GN. Urine of the animals was collected daily and analyzed for its ATP content. Data are shown as mean ± SEM, *n* = 5–8 mice in each group. Data from one representative experiment out of three are shown.

Previously, it has been reported that endogenous ATP can accumulate in extracellular fluids such as sputum or ascites under inflammatory conditions (4, 26, 29). Thus, we first questioned whether the induction of GN in a mouse model would also lead to an accumulation of extracellular ATP in the kidneys, which might be reflected as increased urinary ATP levels in these animals. Indeed as shown in Figure 1, a time-dependent significant increase in ATP-urine levels can be detected in mice with NTS-induced GN compared to vehicle treated control animals.

### Neutralizing ATP-Levels Attenuates GN

**Figure 2:**
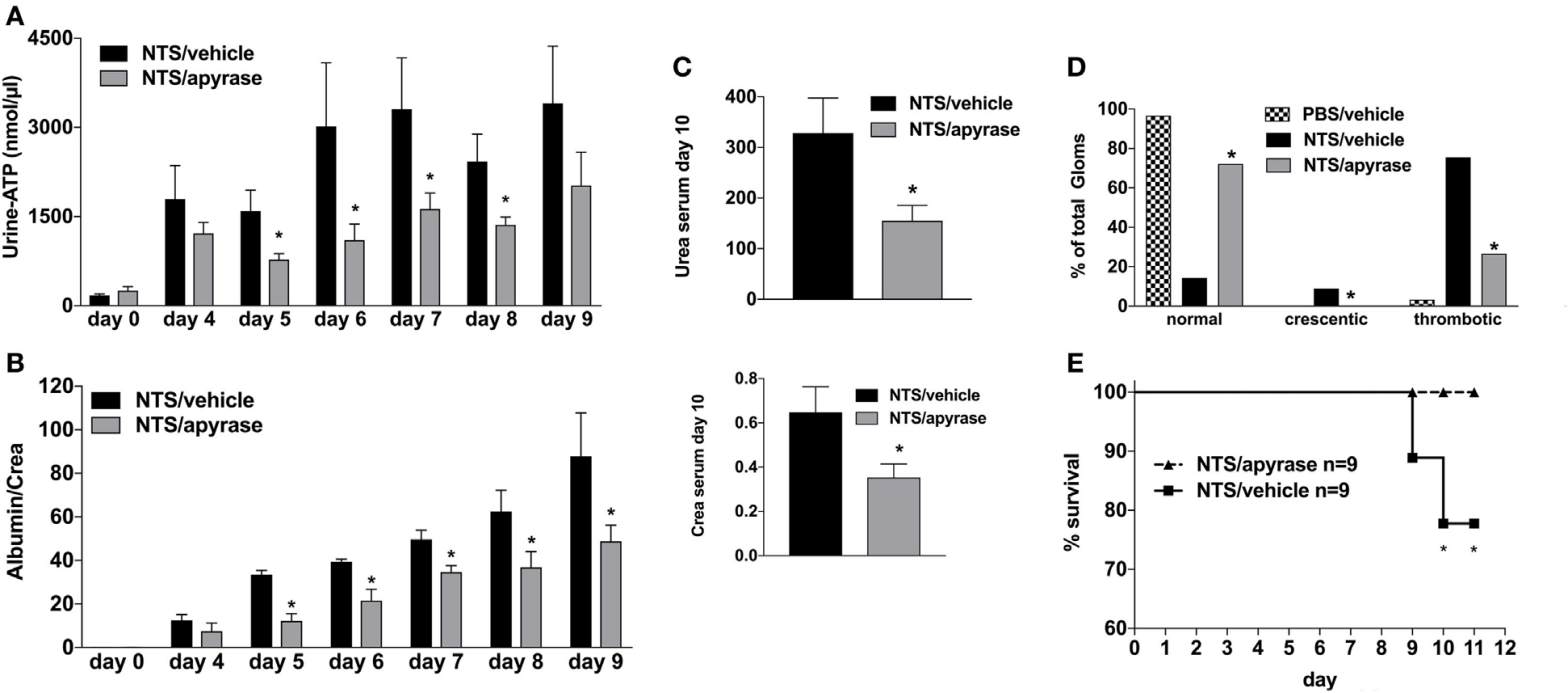
Effect of Apyrase treatment on nephrotoxic serum (NTS)-driven glomerulonephritis (GN). C57BL/6 mice received either vehicle (PBS) or (NTS solved in PBS) intravenous on days 1, 2, and 3 to induce GN. For treatment, they received i.p. injections of either vehicle or apyrase (4 U/ml in 100 µl) on days 1, 3, 5, 7, and 9. Urine was collected daily and analyzed for its adenosine-5’-triphosphate content **(A)** and its albumin–creatinine ratio **(B)**. Animals were killed on day 10 after a blood sample had been taken. This was analyzed for its serum urea and creatinine levels **(C)**. From the killed animals, kidney tissue was prepared and analyzed with microscopy (PAS-staining). Approximately 100 Glomerula in each sample were analyzed following the histological criteria “normal, crescentic, or thrombotic” **(D)**. Follow-up of mice receiving apyrase or vehicle are shown **(E)**. Data are shown as mean ± SEM, *n* = 4–5 mice in each group. *P < 0.05. Data from one representative experiment out of three are shown.

Next, to elucidate the functional relevance of the increased Urine-ATP-levels, animals were treated with the ATP-metabolizing enzyme apyrase or vehicle i.p. at days 0, 2, 4, 6, and 8 (26). Vehicle-treated animals developed all cardinal features of GN including albuminuria, uremia, crescentic glomeruli, and in some animals death due to renal failure occurred (see Figure 2). Treatment of animals with apyrase dramatically decreased urine-ATP-levels, which was accompanied by improved renal function, less proteinuria, decreased histologic glomerular injury scores, and a significant survival benefit compared to vehicle-treated animals (Figures 2A–D).

### Blocking of P2R

**Figure 3:**
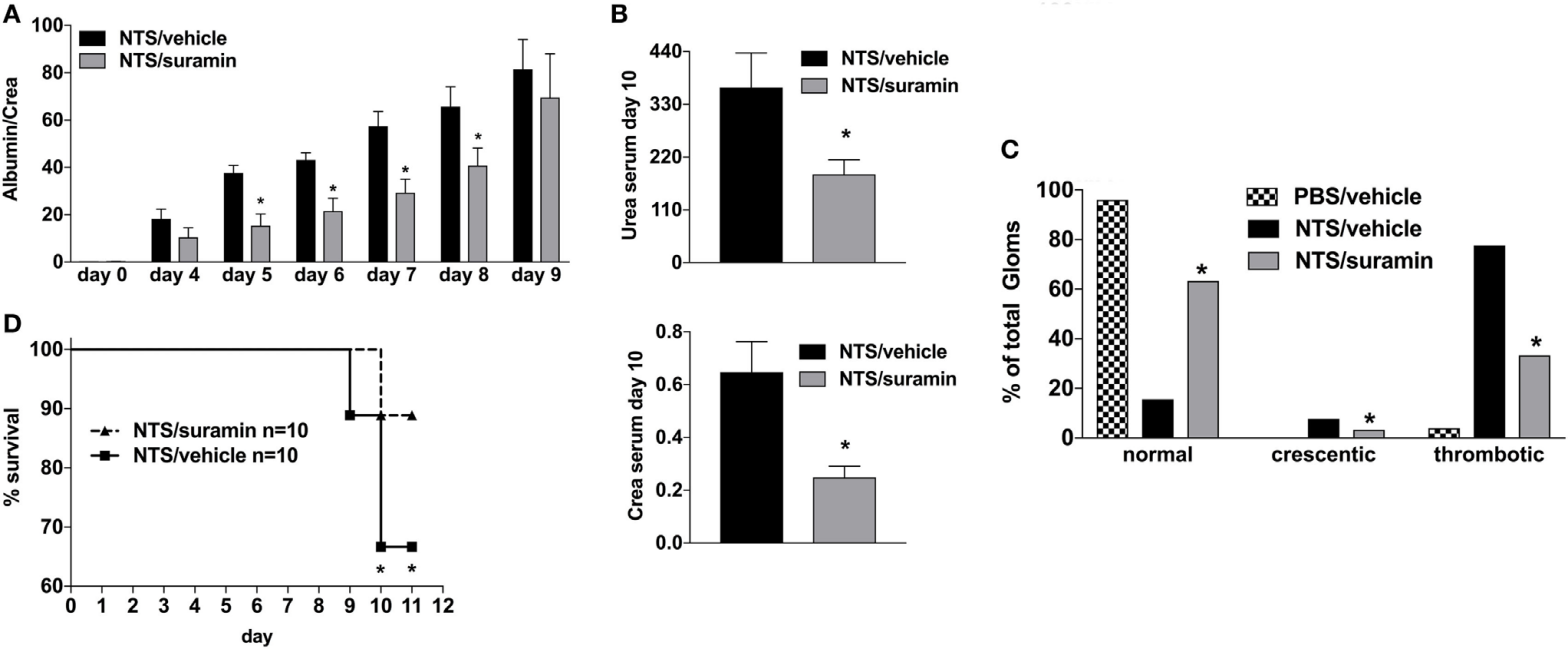
Effect of P2R-antagonist Suramin treatment on nephrotoxic serum (NTS)-driven glomerulonephritis (GN). C57BL/6 mice received either vehicle (PBS) or NTS (solved in PBS) intravenous on days 1, 2, and 3 to induce GN. For treatment, they received i.p. injections of either vehicle or suramin (100 µM in 100 µl) on days 1, 3, 5, 7, and 9. Urine was collected daily and analyzed for its albumin–creatinine quotient **(A)**. Animals were killed on day 10 after a blood sample had been taken. This was analyzed for its serum urea and creatinine levels **(B)**. From the killed animals, kidney tissue was prepared and analyzed with microscopy (PAS-staining). Approximately 100 Glomerula in each sample were analyzed following the histological criteria “normal, crescentic, or thrombotic” **(C)**. Follow-up of mice receiving apyrase or vehicle are shown **(D)**. Data are shown as mean ± SEM, *n* = 4–5 mice in each group. *P < 0.05. Data from one representative experiment out of three are shown.

Activation of the A2A-receptor can reduce NTS and/or lupus-triggered GN (30–32). To rule out that the observed attenuation in apyrase-treated animals is due to an increase of endogenous adenosine and consequent A2AR-acitvation rather than abandoning of ATP/P2R-signaling, experiments with the broad P2R-antagonist Suramin were performed. As demonstrated in Figure 3, the inhibition of P2R by Suramin—given i.p. at days 0, 2, 4, 6, and 8—led to an attenuated NTS-induced kidney damage indicated by less proteinuria and lower creatinine and urea levels at day 10 as well as a decrease in the histological injury score for renal injury at day 10 compared to vehicle-treated animals. Again, these findings were associated with a significant survival benefit.

### Upregulation of Purinergic Receptors in Animals with NTS-Driven GN

**Figure 4:**
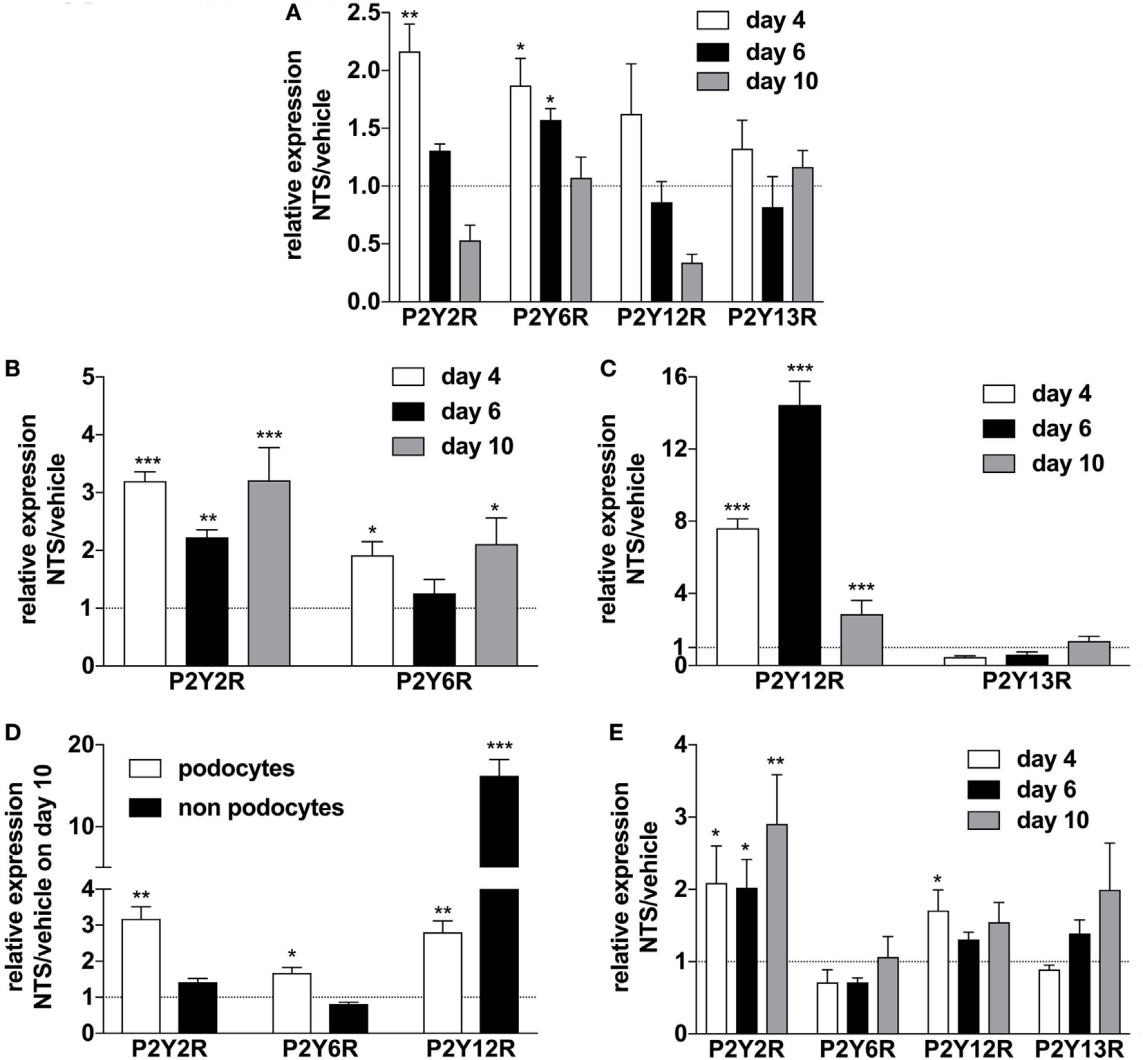
Expression of P2YR subtypes on whole kidney tissue, isolated glomerula, isolated podocytes versus non-podocyte glomerular cells, and blood leukocytes following nephrotoxic serum (NTS)-induced glomerulonephritis (GN). C57BL/6 mice received either vehicle (PBS) or NTS (solved in PBS) intravenously on days 1, 2, and 3 to induce GN. For the isolation of podocytes, we used Gt(ROSA)26Sortm4(ACTB-tdTomato,-EGFP)Luo/J mice, which had been crossed with *hNPHS2*Cre mice. Podocytes of these mice express GFP while all other cells express Tomato. On days 4, 6, and 10 animals were killed after a blood sample for the isolation of leukocytes had been collected. RNA was isolated from whole kidney tissue, isolated glomerula, isolated podocytes, and non-podocytal glomerular cells and from blood leukocytes. Glomerula were isolated using perfusion with magnetic beads. From the isolated glomerula, we separated the GFP-labeled podocytes from non-podoyctes *via* FACS. Relative expression of the different P2Y receptors compared to β2m in triplicates were analyzed using quantitative RT-PCR. **(A)** Expression of P2YR subtypes on whole kidney tissue from *n* = 5 animals per group. **(B,C)** Expression of P2YR subtypes on isolated glomerula from *n* = 5 animals per group. **(D)** Expression of P2YR subtypes on isolated podocytes versus non-podocyte glomerular cells from *n* = 5 animals per group. **(E)** Expression of P2YR subtypes on isolated blood leukocytes from *n* = 5 animals per group. Data are shown as mean ± SEM, *n* = 5 mice in each group. Data from one representative experiment out of three are shown.

In order to elucidate the regulation of purinergic receptors in GN, we analyzed the expression of P2 receptor subtypes in whole murine kidney, isolated glomeruli, isolated podocytes, and “non-podocytes” as well as in blood leukocytes. NTS-driven GN led to a time-dependent upregulation of the P2Y2R, P2Y6R, and P2Y12 subtypes in whole kidney, glomeruli, isolated podocytes, and non-podocytes, respectively (Figures 4A–D). In blood leukocytes, strong upregulation of the P2Y2R and a slightly increased expression of the P2Y12R could be observed (Figure 4E).

### P2Y2R^−/−^ Is Associated With Renoprotection in NTS-Induced GN

The P2Y2R is known to play a crucial role as a “find me signal” for leukocytes in inflammatory disorders (4, 30–32). First, the renal function of naïve P2Y2R^−/−^ mice was compared with WT mice, and as expected, no differences in creatinine/urea serum levels or urine albumin-to-creatinine ratio could be observed (data not shown).

**Figure 5:**
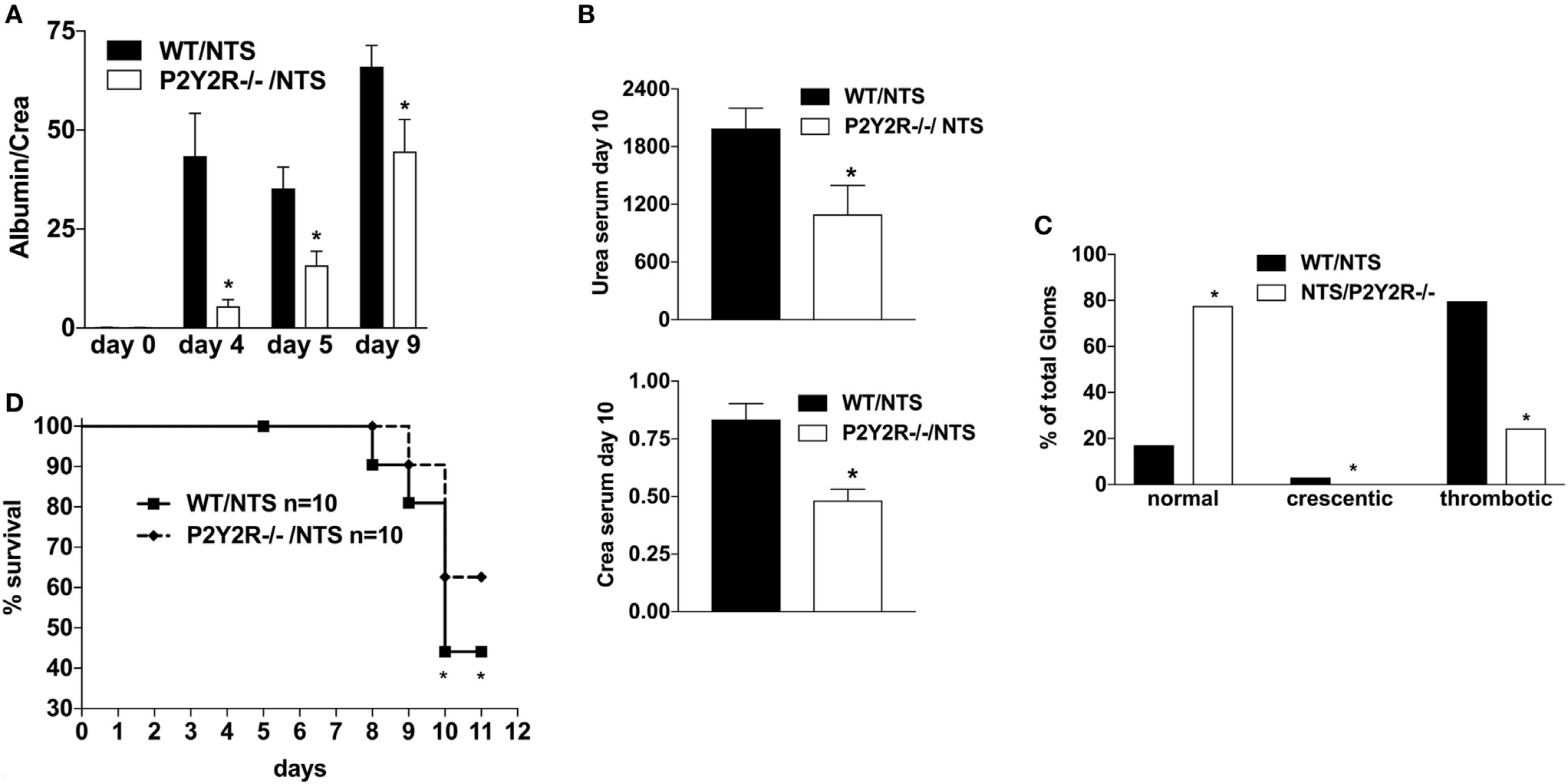
Effect of P2Y2-deficiency in mice with nephrotoxic serum (NTS)-driven glomerulonephritis (GN). P2Y2-receptor^−/−^ mice or wild-type animals (all C57BL/6 mice) received NTS (solved in PBS) intravenously on days 1, 2, and 3 to induce GN. Urine was collected on days 4, 5, and 9 and analyzed for its albumin–creatinine quotient **(A)**. Animals were killed on day 10 after a blood sample had been taken. This was analyzed for its serum urea and creatinine levels **(B)**. From the killed animals, kidney tissue was prepared and analyzed with microscopy (PAS-staining). Approximately 100 Glomerula in each sample were analyzed following the histological criteria “normal, crescentic, or thrombotic” **(C)**. We also performed a survival experiment following the same experimental setup **(D)**. Data are shown as mean ± SEM, *n* = 5 mice in each group. *P < 0.05. Data from one representative experiment out of two are shown.

Next, to assess the potential role of the P2Y2R in the pathogenesis of NTS-GN, P2Y_2_ (^−/−^) mice were treated according to the NTS-driven model of GN. P2Y_2_ (^−/−^) deficiency was associated with a better renal function (less proteinuria, lower creatinine/urea serum levels) (Figures 5A,B), as well as less histological signs of kidney damage and death due to renal failure compared to WT-animals (Figures 5C,D).

### BM-Chimera Revealed a Pivotal Role of P2Y2R-Signaling on Leukocytes in GN

**Figure 6:**
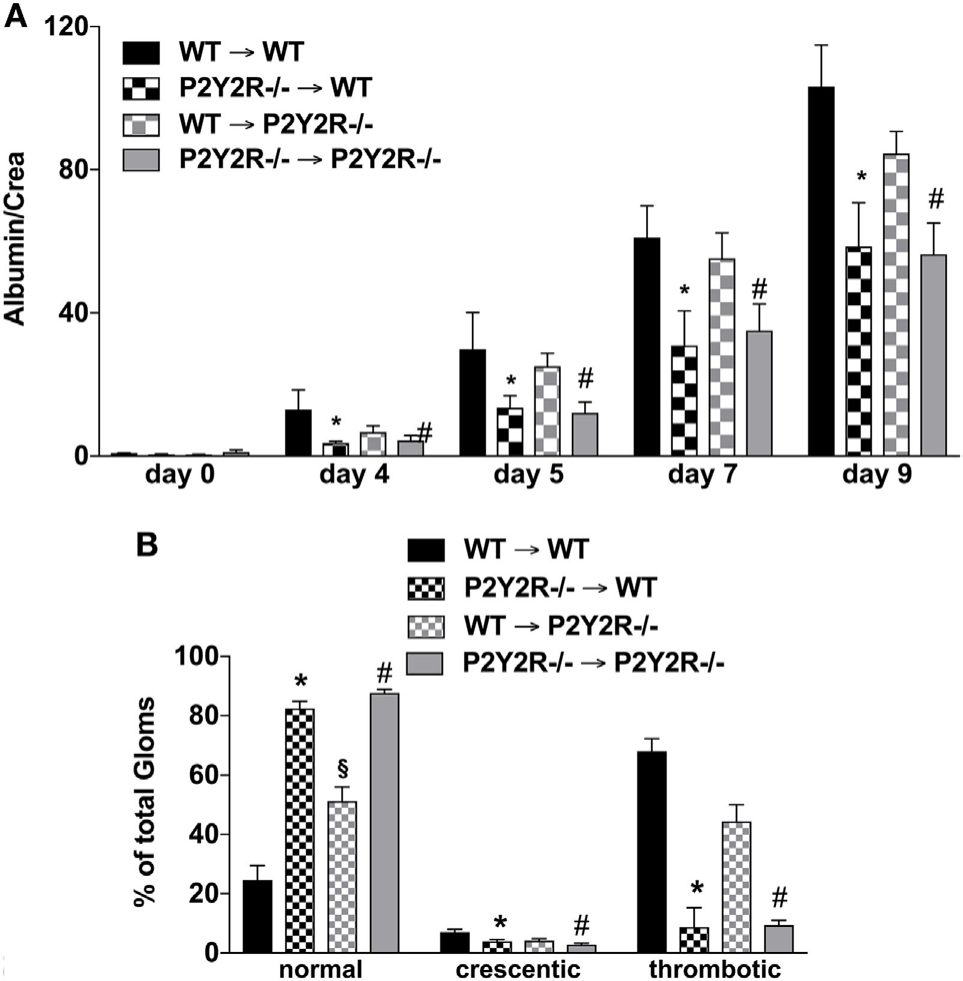
P2Y2^−/−^ deficiency in the haematopetic system is associated with reduced inflammation following nephrotoxic serum (NTS)-induced glomerulonephritis (GN). The different wild-type (WT) and P2Y2R BM-chimera mice received NTS (solved in PBS) intravenous on days 1, 2, and 3 to induce GN. Urine was collected on days 4, 5, and 9 and analyzed for its albumin–creatinine quotient **(A)**. Animals were killed on day 10. From the killed animals, kidney tissue was prepared and analyzed with microscopy (PAS-staining). Approximately 100 Glomerula in each sample were analyzed following the histological criteria “normal, crescentic, or thrombotic” **(B)**. Data are shown as mean ± SEM, *n* = 5 mice in each group. *P < 0.05. P2Y2R^−/−^ in P2Y2R^−/−^ versus WT in WT. ***^#^****P* < 0.05. P2Y2R^−/−^ in WT versus WT in WT. Data from one representative experiment out of two are shown.

To distinguish the role of immune and mesenchymal cells in the P2y2R-driven kidney damage in GN, P2Y2R^−/−^ and WT BM chimeras were challenged with NTS. As shown in Figure 6, P2Y2R deficiency of the donor (P2Y2R^−/−^ = > wt) displayed a significant decrease in all cardinal features of GN, as evidenced by reduced proteinuria, and reduced “damage in histology.” In contrast, P2Y2R^−/−^ animals reconstituted with WT hematopoietic system (recipient) (wt = > P2Y2R^−/−^) showed only a slight reduction in histological scores compared to WT animals.

## Discussion

In the current manuscript, we investigated the role of the P2Y2R in the course of NTS-induced GN in mice. GN is one of the leading causes for end-stage kidney disease. Especially, the rapid progressive form has both an unfavorable outcome and is difficult to treat with prominent non-renal side-effects. We used NTS to induce an anti-basement-membrane-dependent GN, which leads to rapid loss of kidney function as seen by massive proteinuria, an increase in serum creatinine levels, and is followed by death within 8–10 days. Here, we could demonstrate that the induction of NTS-induced GN is associated with a surge of urinary ATP levels. Of note, ATP neutralization or unspecific purinergic blockade markedly reduced the development of NTS-mediated GN in mice. Mechanistically, we provide evidences that the observed effects might involve the activation of the P2Y2R on leukocytes. In summary, our data suggest that extracellular ATP—*via* binding to purinerigic receptors such as P2Y_2_R—is involved in the pathogenesis of antibody-mediated GN.

In healthy tissues, the extracellular ATP concentration is kept in the low nanomolar range due to tight control by ubiquitous ecto-nucleotidases, which dephosphorylate ATP to AMP and adenosine. However, due to the high intracellular ATP concentrations (5–8 mM), a steep transcellular ATP gradient can occur (3, 4). Thus, it is not surprising that multiple cells release ATP upon cellular stress or injury, such as mechanical stress, hypoxia, or inflammation causing a massive accumulation of extracellular ATP (up to micromolar levels) (2–4). Notably, we observed a time-dependent strong increase in urinary ATP concentrations in NTS-induced GN. Increased extracellular ATP levels have also been found in the interstitial fluid following BP-induced changes in renal vascular resistance (33) as well as in cyst fluid in autosomal dominant cystic kidney disease (34, 35). The reason for the increased ATP concentrations could be either due to an increase of ATP release or due to suppression of ATP-degrading ecto-nucleotidases. Indeed, a decrease in ecto-nucleotidase expression has been observed in kidney tissue following ischemic reperfusion injury (36) or after the stimulation with reactive oxygen species (ROS) (37), which are known to play a pivotal role in the pathogenesis of chronic (inflammatory) kidney diseases including GN (38, 39). In contrary to this assumption, in our model, we could detect a “compensatory” upregulation of ectonuecleotidase CD39 expression in the whole kidney as well as in glomeruli and podocytes in mice with NTS-mediated GN (see Figure S1 in Supplementary Material). These findings are in line with recent observations in patients and mice with cigarette-smoke induced pulmonary inflammation (29). One could argue that the increased urinary ATP levels merely reflect the degree of tissue damage and do not have pathogenetic implications in inflammation or tissue destruction. However, our data show that systemic treatment of animals with apyrase—which metabolizes ATP/ADP to AMP (40, 41)—leads to a reduction in urinary ATP-levels, *via* neutralization of kidney ATP levels, which was associated with a decreased severity of NTS-mediated GN.

Beside the expression of CD39 in glomeruli also the expression of CD73, which degrades AMP to adenosine, is slightly upregulated during GN. Garcia reported that endogenous adenosine *via* A2aR signaling can attenuate immune-associated inflammatory kidney diseases in rats (30). Thus, one could assume that the protective effect of apyrase could be driven by an increase of adenosine-A2AR signaling. However, our experiments using the broad P2YR antagonist suramine clearly demonstrate that blocking the ATP/P2R-pathways can suppress features of antibody-mediated GN. With all limitations of such animal models, these data support the hypothesis that purinergic receptor signaling may play a role in the pathogenesis of antibody-mediated GN. Further studies should aim to confirm an involvement of P2YR signaling in human RPGN.

Once in the extracellular space ATP can bind to P2Y1, P2Y2, P2Y4, P2Y12, P2Y13R and in human cells also to the P2Y11R. Interestingly, depending on its concentration, ATP can thereby also act as a partial agonist for P2Y1R and P2Y13R or even as an antagonist for the P2Y4R or P2Y12R. This is correct for most P2YR beside the P2Y2R, where only ATP acts as an agonist (4, 42).

Importantly, recent studies could demonstrate that ATP released by activated inflammatory cells *via* connexin or pannexin hemichannels (43, 44) and concomitant P2Y2 signaling serves as a “find me signal” for leukocytes promoting phagocytic-clearance of apoptotic cells or bacteria, thereby contributing to the resolution of inflammation (7, 45, 46).

On the other hand, a pivotal role of the ATP/P2Y2R axis in the migration and chemokine production by leukocytes has been demonstrated (45, 47). Indeed, we could recently show that endogenous released ATP *via* activation of P2Y2R on leukocytes contributes to the pathogenesis of inflammatory diseases such as asthma, lung fibrosis, hepatitis, atherosclerosis, or GvHD (23, 48–51). The ambivalent behavior (“friend or foe”) of the P2Y2 receptor during inflammatory disease states also applies for inflammatory kidney diseases. For instance, Potthoff et al. reported that deficiency of P2Y2R is linked to greater inflammation and renal injury in a mouse model of subtotal-nephrectomy chronic kidney diseases (19). Furthermore, P2Y2R, *via* regulating IL-6 and IL-8 release from renal epithelial cells, might contribute to innate immune responses against *Escherichia. coli* bacterial urogenital infection (52, 53). In contrary, P2Y2R signaling has been assumed to contribute to the development of mesangial proliferative GN or lithium-induced nephrogenic diabetes insipidus (15, 54, 55) as well as to the progression of polycystic kidney diseases (56).

In our model of NTS-driven GN, we observed a slight transient (day 4 and day 6 after NTS-injection) upregulation of the P2Y2R expression in kidney tissue of animals with GN. Interestingly, an even higher and persistent upregulation in isolated glomeruli could be found. Mechanistically, this could be due to the recruitment of M2 macrophages around glomeruli, which may restrict inflammation to a certain area and maintain tubular function (57). In support, qtPCR with blood leukocytes from these animals revealed a strong upregulation of P2Y2R, suggesting an involvement of P2Y2R in the pathogenesis of GN. Of note, a transient upregulation of P2Y2R in isolated glomeruli has been previously shown in anti-Thy1-driven mesangial proliferative GN in rats (15).

The hypothesis that P2Y2R signaling (on hematopoietic cells) contributes to the development of GN is supported by our finding that P2Y2R deficiency is linked to a milder phenotype of NTS-mediated GN. Furthermore, experiments with chimera (P2Y2R^−/−^ and wt animals) revealed that the expression of P2Y2R on hematopoetic cells plays the major role for this effect while P2Y2R^−/−^ mice reconstituted with wt bone marrow only showed a small albeit significant reduction in the number of thrombotic glomeruli in comparison to wt animals.

Mechanistically, as mentioned above, activation of the P2Y2R by endogenously released ATP—as a “find my signal”—is pivotal for the recruitment of leukocytes such as neutrophils, dendritic cells, and macrophages to the side of inflammation (4, 45–47). Interestingly, macrophages are important effector cells in the pathogenesis of GN (58). For instance, accumulation of blood macrophages is linked closely to the severity of glomerular injury in a NTS-driven model in rats (59). Furthermore, ATP is a powerful macrophage stimulant, which *via* P2 × 7R-signaling induces the secretion of cytokines such as IL-1β and IL-18 (4), both are involved in crescent formation and tubulointerstitial injury (60, 61). Not surprisingly, mice lacking P2 × 7R displayed a milder phenotype in antibody-mediated GN (13). In accordance, we detected a reduced number of CD68+ macrophages in glomerular cross-sections from P2Y2^−/−^ mice compared to WT animals (data not shown). Beside macrophages also neutrophils and DC have recently been implicated to play a role in the pathogenesis of GN (62–64). Of note, beside migration, P2Y2R-activation on leukocytes triggers the release of various pro-inflammatory mediators such as cytokines, NE, or ROS (4, 25, 65). Notably, an involvement of ROS in the pathogenesis of GN has been previously implicated (38, 39).

As suramin blocks several P2Y-purinergic receptors, an involvement of multiple purinergic receptors in the pathogenesis of GN could be assumed. Indeed, Hohenstein and colleagues reported that P2Y1R^−/−^ deficiency is renoprotective in a mouse model of antibody-driven crescentic GN (14). The strong upregulation of P2Y2R in blood leukocytes and of P2Y2R, P2Y6R, P2Y13R in the kidney, glomeruli, or podocytes respectively, as well as the experiments using P2Y2R^−/−^ mice suggest that this receptor subtype might be primarily involved. Thus, further investigation is needed to identify the specific subtypes of particular relevance in the pathogenesis of antibody-mediated GN to test the potency of selective purinergic receptor antagonists in this model. Subsequently, translation of preclinical findings into human trials is paramount, as the current mainstay of therapy relies on strong immunsuppressive medication together with antibody removal, which despite all side effects only generates a moderate outcome (66, 67). Hence, new targets and avenues for treatment, amelioration, and relapse prevention of RPGN are an unmet clinical need.

Taken together, the present findings suggest that enhanced endogenous ATP concentrations, *via* activation of purinergic receptors such as P2Y2R, might be causally related to the pathogenesis of antibody-mediated renal damage and might be a new target for the therapy of GN.

## Ethics Statement

All experiments were approved by the local animal ethics committee (Regierungspraesidium Freiburg).

## Author Contributions

Experiments were planned, performed, and analyzed by LR, SZ, LS, BH, KA, CM, RZ, TH, MI, and FG. SC, AZ, and MI helped with the analysis of the experiments. The manuscript was written by LR, TH, FG, and MI. The manuscript was read and approved by all authors.

## Conflict of Interest Statement

The authors declare that the research was conducted in the absence of any commercial or financial relationships that could be construed as a potential conflict of interest.
