# Supplementary figures and images for: P2Y2R Signaling Is Involved in the Onset of Glomerulonephritis
Source: Front Immunol. 2018 Jul 16;9:1589. doi: 10.3389/fimmu.2018.01589 (PMC6054981; doi:10.3389/fimmu.2018.01589)

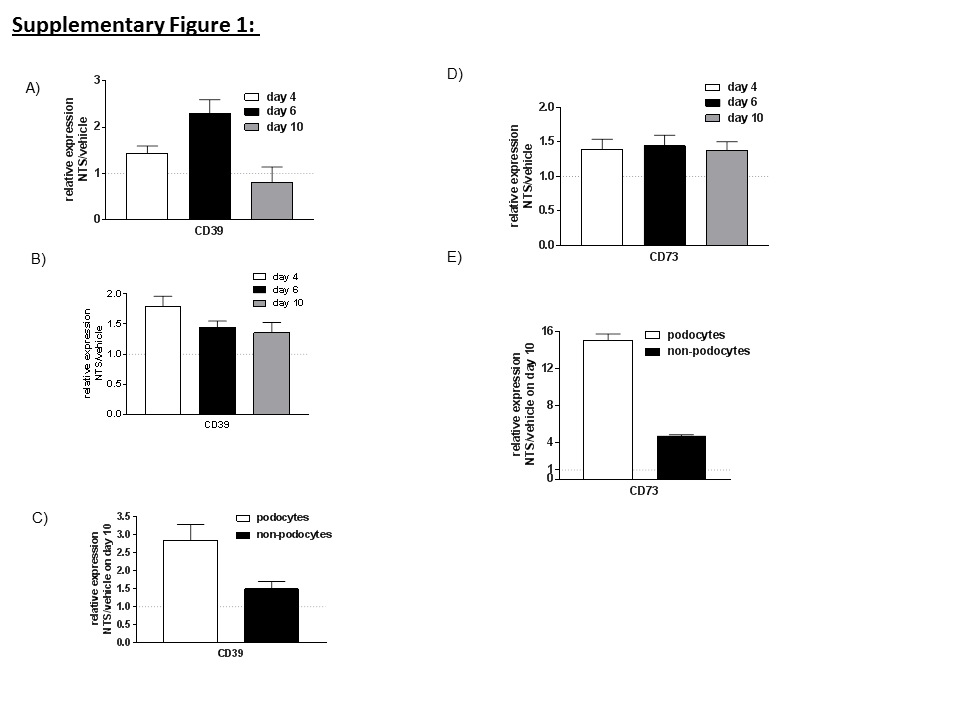

Supplement: Supplementary file 1 [file image_1.tif]
